# The loss of flight in ant workers enabled an evolutionary redesign of the thorax for ground labour

**DOI:** 10.1186/s12983-020-00375-9

**Published:** 2020-10-19

**Authors:** Christian Peeters, Roberto A. Keller, Adam Khalife, Georg Fischer, Julian Katzke, Alexander Blanke, Evan P. Economo

**Affiliations:** 1 Institut d’Écologie et des Sciences de l’Environnement, Sorbonne Université, CNRS, 75005 Paris, France; 2 Museu Nacional de História Natural e da Ciência & cE3c-FCUL, Universidade de Lisboa, Lisbon, Portugal; 3 Biodiversity and Biocomplexity Unit, Okinawa Institute of Science and Technology Graduate University, Onna, Okinawa, 904-0495 Japan; 4 Institute for Zoology, Biocenter, University of Cologne, 50674 Cologne, Germany

**Keywords:** Formicidae, Mutillidae, Queen, Micro-CT, Muscles, Endoskeleton, Central place foraging

## Abstract

**Background:**

Explanations for the ecological dominance of ants generally focus on the benefits of division of labour and cooperation during foraging. However, the principal innovation of ants relative to their wasp ancestors was the evolution of a new phenotype: a wingless worker caste optimized for ground labour. Ant workers are famous for their ability to lift and carry heavy loads, but we know surprisingly little about the morphological basis of their strength. Here we examine the consequences of the universal loss of flight in ant workers on skeletomuscular adaptations in the thorax for enhanced foraging on six legs.

**Results:**

Using X-ray microcomputed tomography and 3D segmentation, we compared winged queens and wingless workers in *Euponera sikorae* (subfamily Ponerinae) and *Cataglyphis savignyi* (subfamily Formicinae). Workers are characterized by five major changes to their thorax: i) fusion of the articulated flight thorax (queens) into a rigid box optimized to support the muscles that operate the head, legs and abdomen, ii) redesign of internal cuticular structures for better bracing and muscle attachment, iii) substantial enlargement of the neck muscles for suspending and moving the head, iv) lengthening of the external trochanter muscles, predominant for the leg actions that lift the body off the ground, v) modified angle of the petiole muscles that are key for flexion of the abdomen. We measured volumes and pennation angles for a few key muscles to assess their increased efficacy. Our comparisons of additional workers across five genera in subfamilies Dorylinae and Myrmicinae show these modifications in the wingless thorax to be consistent. In contrast, a mutillid wasp showed a different pattern of muscle adaptations resulting from the lack of wing muscles.

**Conclusions:**

Rather than simply a subtraction of costly flight muscles, we propose the ant worker thorax evolved into a power core underlying stronger mandibles, legs, and sting. This contrasts with solitary flightless insects where the lack of central place foraging generated distinct selective pressures for rearranging the thorax. Stronger emphasis is needed on morphological innovations of social insects to further our understanding of the evolution of social behaviours.

## Background

Ants are among the most abundant terrestrial animal groups, and the benefits of division of labour within colonies is thought to be the central factor in their ecological success. Ants stand out from social bees and wasps because their workers are universally flightless. This striking morphological differentiation between castes avoids trade-offs and optimizes for ground abilities. Ant reproductives (queens and males) need to fly when they are young, but workers are freed from the need to find mates and disperse. Instead, ant workers are famous for their abilities to lift and carry objects [1–5], suggesting adaptations for enhanced foraging on six legs. The morphology underlying those enhanced abilities, however, remains unclear.

All social insects are central place foragers—food is brought back to the nest instead of being eaten on the spot—and the physical ability to retrieve comparatively large amounts of food is paramount [6]. While the workers of social bees and wasps bring back their food by flight, ant workers transport food by walking. Importantly, ant workers are strong enough to lift and carry aloft prey and other food items, unlike various solitary predatory wasps that drag prey when too large to retrieve by flight. Foraging efficiency is also impacted by speed of walking, while running is essential to escape from predators (in contrast, wasps and bees escape by flight). Moreover, the abdomen can serve to hold food reserves, while deftness of its tip is essential in predation and defense to sting or spray formic acid (The sting has its own set of muscles for extrusion and retraction).

The thorax (known as ‘mesosoma’ in Apocritan hymenopterans because it includes the usual three segments fused with the first true abdominal segment, called *propodeum* [7–9]) bears the wings and legs, and it supports the head and abdomen, hence it is subjected to often-conflicting mechanical constraints. Keller et al. (2014) [10] identified a shift in the relative size of the external dorsal plates (where muscle fibres attach) in the anterior thorax as a critical difference between winged queens and flightless ant workers. Reduction of the *mesonotum* (dorsal plate of the second thoracic segment, T2) and concomitant expansion of the *pronotum* (dorsal plate of T1) in workers reflect a trade-off: elimination of flight-related structures allows increased neck muscles in the prothorax. However, the posterior external plates vary little between queens and workers, and it remained unclear if there is also remodelling of the endoskeleton and muscles transmitting force to the legs and abdomen. Among the Hymenoptera, only ant workers and a few solitary parasitoids (e.g. females of Chyphotidae, Mutillidae, and Tiphiidae) are wingless. The anatomy of the wingless thorax was investigated early on by Lubbock (1881) and Janet (1907) [7, 11], described externally by Tulloch (1935) and Reid (1941) [9, 12], and recently described using 3D reconstruction [13]. Despite this accumulation of data over more than a century, the morphological consequences of wing loss have hardly been investigated.

Winglessness in ant workers has been considered a reduction to facilitate mobility in cramped environments [6], without examining consequences on thorax architecture. We hypothesized that the evolution of a worker caste freed from the anatomical and functional constraints of flight is a critical innovation that allowed the remodelling of the thorax for ground labour. Using X-ray microcomputed tomography (micro-CT), we performed detailed segmentation to compare conspecific workers and winged queens in two distantly related exemplar genera, *Euponera* (subfamily Ponerinae) and *Cataglyphis* (Formicinae). We show significant fusion of external plates and modifications of endoskeleton, neck, leg, and petiole muscles. We confirmed this in a phylogenetically broad sample of ant genera (Table 1). We discuss that ant workers have a distinct thorax relative to other wingless insects (including solitary wingless hymenopterans), and explain this as a function of sociality and living in perennial nests: central place foraging selects for optimal gathering of food.

**Table 1:** Thorax modifications resulting from the lack of wing muscles in additional taxa from three ant subfamilies (Dorylinae, Formicinae, Myrmicinae) and a female mutillid wasp (subfamily Mutillinae, tribe Smicromyrmini or Trogaspidiini) examined by micro-CT and SEM

Subfamily	Species	Fusion of external plates (SEM)?	Angle of profurca platform?	Origin of direct head muscle Idlm1?	Indirect muscle Idvm5 enlarged?	Origin of mid- & hindlegs trochanter muscles?	Enlarged petiole muscles?
Formicinae	*Colobopsis truncata*	articulated pronotum and mesonotum	more vertical	mesonotum roof	pronotum larger	mesonotum roof	entire propodeum roof
Dorylinae	*Dorylus wilverthi*	promesonotal fusion	more vertical	mesonotum roof	pronotum larger	mesonotum roof	entire roof
Myrmicinae	*Carebara perpusilla*	promesonotal fusion	more vertical	absent ^a^	pronotum larger	mesonotum roof	entire roof
Myrmicinae	*Melissotarsus beccarii*	promesonotal fusion	more vertical	absent ^a^	extremely large	mesonotum roof	highly reduced ^a^
Myrmicinae	*Messor barbarus*	promesonotal fusion	more vertical	mesonotum roof	pronotum larger	mesonotum roof	entire roof
Myrmicinae	*Pheidole oculata*	promesonotal fusion	more vertical	mesonotum roof	pronotum larger	mesonotum roof	entire roof
Mutillinae (Mutillidae)	unidentified	promesonotal fusion	tilted back	absent^b^	no, smaller than petiole	mesofurca for midlegs	past midlegs only

^a^associated with miniaturisation

^b^linked to hypognathy

## Methods

### Taxon selection

We focused on two species with workers of similar body sizes, belonging to distantly related ant subfamilies. *Euponera sikorae* is a generalized species in the subfamily Ponerinae, and a good example of the lowest queen-worker dimorphism in ants, likely to represent the ancestral condition [14]: the thorax of workers remains generally stout, with parallel sides and an uninterrupted dorsum (Additional file 1). *Cataglyphis savignyi* is an exemplar of the large subfamily Formicinae, and illustrates a pronounced level of queen-worker dimorphism [15]: the thorax of workers is slender, especially in its middle (Additional file 2). Head width of workers varies from 1 to 3 mm (N. Lecocq de Pletincx pers. comm.), and we scanned an individual similar in body size to the queen.

The most recent common ancestor of *Cataglyphis* and *Euponera* is a deep node in the ant phylogeny [16]. Thus, it is likely that any similarity in thorax design between these two taxa is shared by most ants. Our primary analysis concentrates on two species because micro-CT analysis is labour-intensive. However, we have examined scans of workers from a number of genera across the ant phylogeny (Table 1) to verify that the changes we describe are general features of the ant thorax. We also examined the thorax of a solitary Mutillid wasp, representing an independent loss of flight within the Hymenoptera.

*Euponera sikorae* was collected in Madagascar (Zahamena NP, Toamasina province); *Cataglyphis savignyi* in Israel (Arad Park); subfamily Mutillinae (tribe Smicromyrmini or Trogaspidiini) in Uganda (Kanyawara Biological Station, Kibale).

### Specimen preparation and micro-CT scanning

Micro-CT scans were carried out at OIST using a Zeiss Xradia 510 Versa 3D X-ray microscope. Ant specimens were stored in 90% ethanol, then stained in a 2 M iodine solution for a minimum of 24 h and transferred into pipette tips filled with 99% ethanol before scanning. Scan settings such as current, voltage, and exposure were adjusted for each specimen to yield maximum scan quality. Full 360 degree rotation resulted in 1601 projections. Scans had resolutions of 993 × 1013 × 988 pixels (vertical stitching increased this to 3159 / 3514 × 1013 × 988 pixels for *Euponera* and *Cataglyphis* queens, respectively). Post-imaging 3D reconstruction was performed using the Zeiss Scout-and-Scan Control System Reconstructor software (version 11.2) and output files were saved in DICOM format.

### Cuticle thickness measurements

Scan files were loaded into Drishti 2.6.5 [17] to generate 3D models. Two ‘Clipping Planes’ were positioned at a 90° angle across pronotum and propodeum to get the most accurate measurements of cuticle thickness. For every plane, a ‘Viewport’ was added to visualize the section, and ten measurements were made on each section (clockwise, numbered from 1 to 10; Additional file 3) using the ‘Path’ function.

### Segmentation

We assessed homologies of muscles and sclerites relative to *Apis mellifera* [18] in which both workers and queens are winged. Muscle nomenclature follows Friedrich & Beutel 2008 and Liu et al. 2019 [13, 19]: for example, Idvm5 denotes a dorsoventral muscle in T1 inserting on the propleura (ventral plate), while IIscm6 is a sterno-coxal muscle in T2 inserting on the trochanter.

We combined Amira 6.3.0 and ITK-SNAP 3.6.0 [20], with manual segmentation every five to ten slices followed by either the ‘Interpolation’ tool in both softwares or the ‘region competition’ algorithm for semi-automatic segmentation in ITK-SNAP. Subsequently, we manually cleaned the edges of automatically segmented structures, in particular between neighbouring muscles that are difficult to recognize as separate structures by the algorithm.

### Muscle volume measurements

Muscle segmentations were exported as mesh files (.stl) from ITK-SNAP into the software Blender 2.81 (www.blender.org). Spaces between fibres result from shrinkage during ethanol preservation, and we integrated over entire muscle segmentations. After scaling using voxel size, we fitted a 3D object (usually an Icosphere) to each muscle mesh. We achieved good fit using (1) the “subsurface” modifier on the 3D object to create more polygons and (2) the “shrinkwrap” modifier to fit this 3D object around the muscle mesh. We measured the volume of this 3D object and used it as the physiological muscle volume. In case of a poor fit, we split the muscle mesh into two meshes of simpler shapes and fitted 3D objects separately. Volumes were then normalized (Additional file 9) using the volume of the inner thorax which was segmented with Amira by selecting the space enclosed by cuticle, after closing the openings for head, leg and petiole insertions.

### Fibre tracing and angle measurement

Vertebrate muscles have mostly parallel fibres between points of attachment (e.g. bone extremities), thus measuring physiological cross-sectional area (PCSA) is meaningful to infer muscle force. In contrast, insect locomotory muscles can ‘spread out’ as they increase in size, i.e. additional fibres attach to a greater area of the endo- and exoskeleton, then converge to a tendon. Hence, pennation angles are more relevant to distinguish between efficient and less efficient fibres. We selected segmented petiole muscles of *Euponera* to compare pennation in queen and worker. We traced individual fibres with Amira XTracing 2019.2 and used Blender 2.81 to calculate the angle between each fibre and the direction of force production of the respective muscle (Katzke et al. in prep.). In this approach, the coordinate system defined by the micro-CT image stack is utilized to treat orientations of anatomical features as vectors.

## Results

While ant queens retain the ancestral thorax morphology of flying Hymenoptera, we identified five major deviations of the worker thorax involving the exoskeleton, endoskeleton, neck muscles, leg muscles, and petiole muscles. Although our discussion focuses on *Cataglyphis* and *Euponera*, these modifications are present across all the ant workers examined (Table 1). Together, they represent the evolution of an enhanced power core for more effective foraging on six legs.

### Fusion of the flexible articulated thorax into a rigid thoracic box

**Fig. 1:**
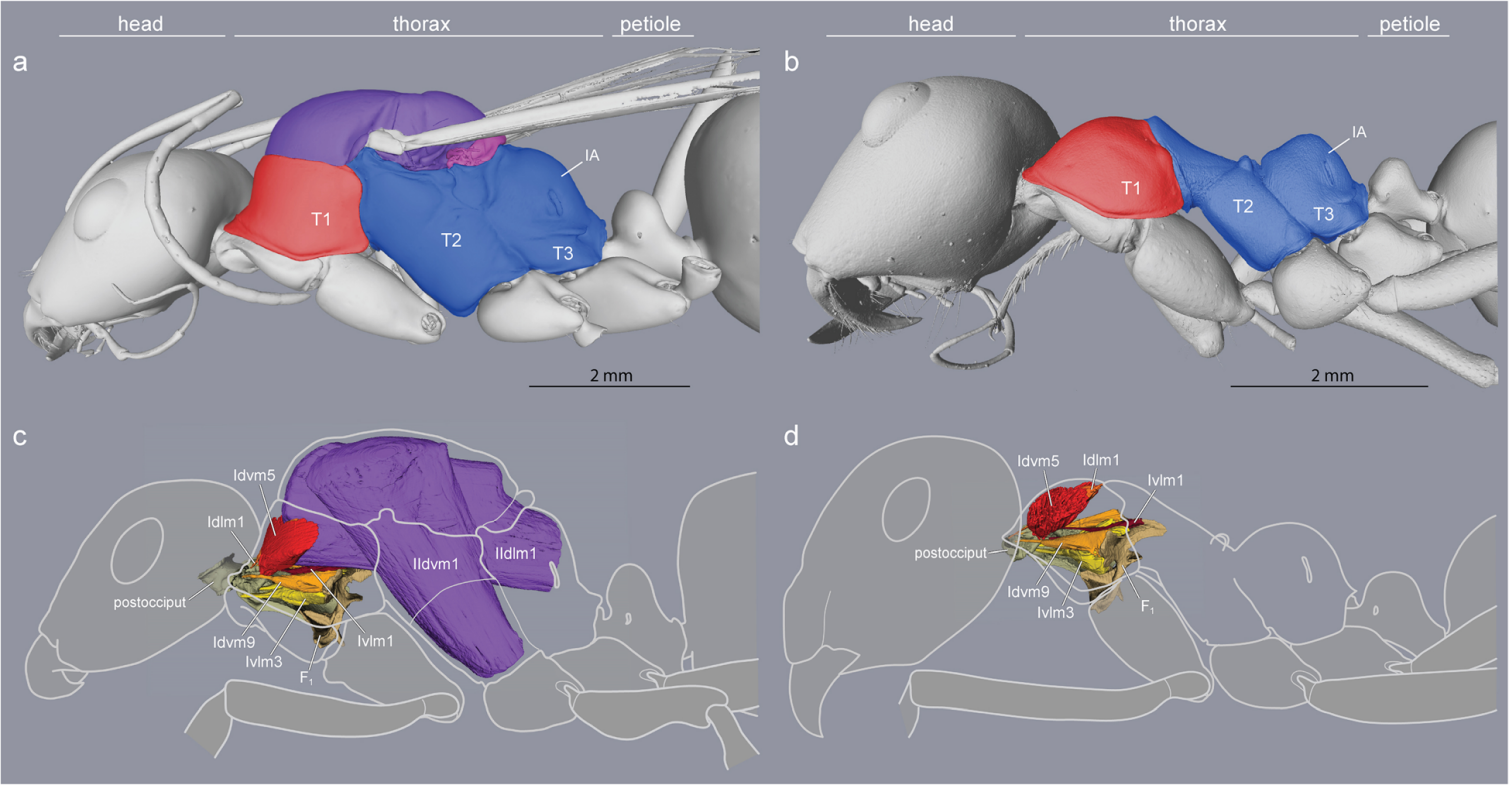
Caste differences in fusion of thoracic plates and presence/absence of wing muscles (*Cataglyphis savignyi*). **a** A large mesonotum (purple) in queens freely articulates dorsally, and **c** carries huge wing muscles (purple) that occupy most of the thoracic cavity. **b** The mesonotum in workers is much reduced and fully fused with the posterior section of the thorax to form a rigid box-like structure (blue). **d** Neck muscles in workers (red, orange, yellow) expand in the absence of wing muscles within the enlarged anterior thoracic cavity (B, red). T1, prothorax. T2, mesothorax. T3, metathorax. IA, propodeum

**Fig. 2:**
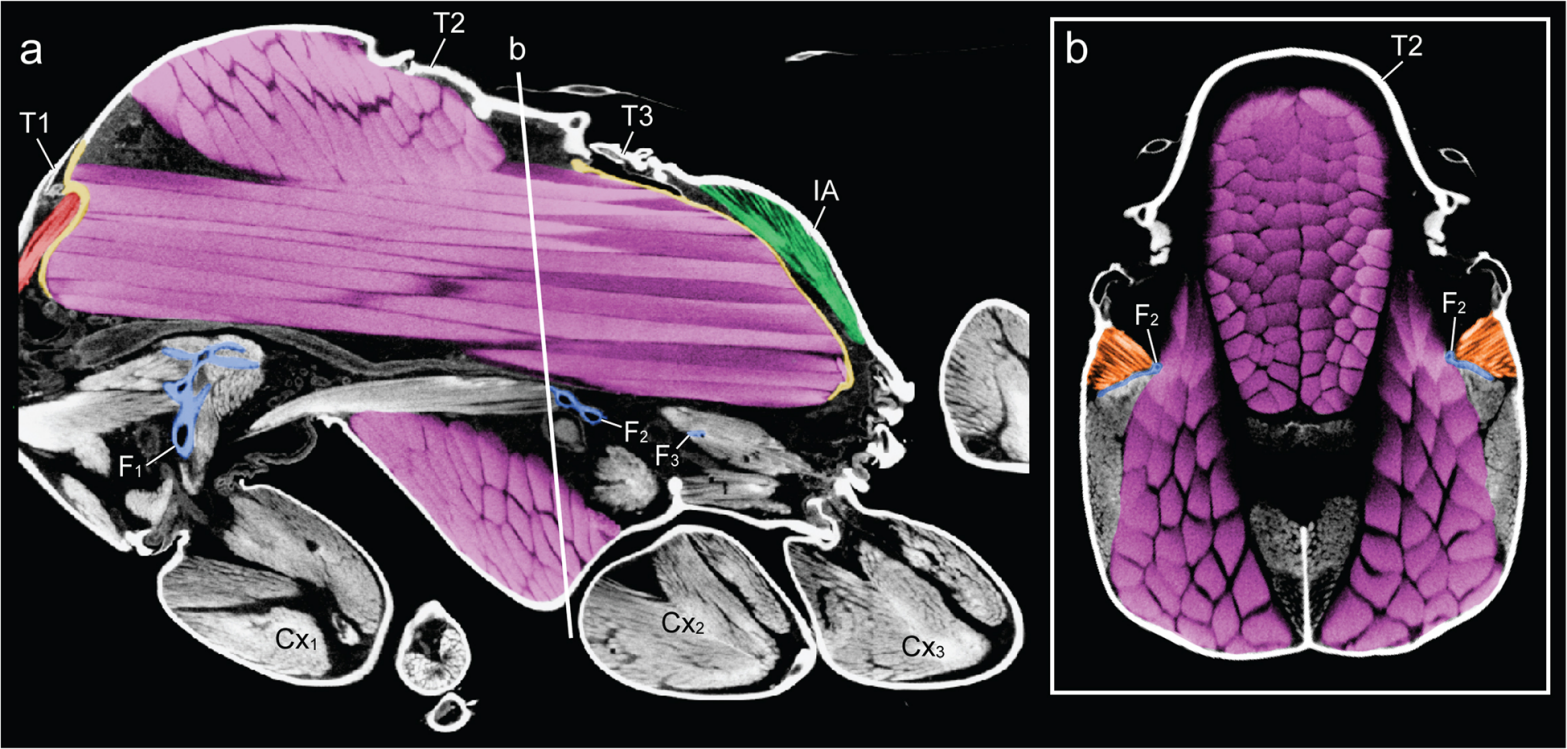
Constraints of the wing muscles (purple) on the height of the furcae (blue) and volumes of neck (red) and petiole (green) muscles in thorax of *Cataglyphis savignyi* queen. **a** Sagittal section from micro-CT scan. Note the anterior and posterior phragmata in yellow. **b** Transverse section through mesothorax, showing the muscular connection (orange) between the mesofurca (blue) and the lateral thoracic walls. T1–3, pro-, meso-, and metathorax respectively. IA, propodeum. F1–3, pro-, meso-, and metafurca respectively. Cx1–3, fore-, mid-, and hindcoxa respectively

The thorax of a winged insect is essentially a flying engine with powerful wing muscles that vibrate articulated external plates called *sclerites*. As in other winged Hymenoptera, ant queens have huge indirect wing muscles (41 and 52% of thorax volume in *Euponer*a and *Cataglyphis*, respectively) that fill the prominent mesothorax (T2) but also adjacent segments (Figs. 1c and Fig. 2). This is because the longitudinal muscles are attached to flexible cuticular invaginations (*phragmata*) of the mesonotum that extend into both the prothorax (T1) towards the front and the propodeum (IA) towards the back (Fig. 2; the metathorax, T3, is much reduced dorsally and only serves for insertion of the hindwings [8]). Hence, much of the relatively small cavity of the prothorax of ant queens is occupied by wing muscles, with the neck muscles squeezed against the exoskeletal outer wall. Flapping of the wings is achieved not by direct muscle action, but by vibration of the entire thoracic box caused by the indirect wing muscles that deform the dorsal plates to which the wings are attached [21]. Flexibility of the thorax and free articulation of the dorsal plates (Fig. 1a) are thus essential for flying, and winged ant queens conform to the general groundplan of Hymenoptera.

Ant workers lack wing muscles and the corresponding phragmata, and the plates of T2, T3 and IA are fused into a single rigid structure forming the posterior two-thirds of the thorax (Fig. 1b, Additional file 1). The first third is the prothorax (T1) housing all the muscles that move the head, and it is greatly enlarged dorsally [10].

Thickness of *Euponera* cuticle is about three times that of *Cataglyphis*, in both queens and workers (Additional file 3). Thickness varies according to dorsal or lateral position of measurements within one segment, and variability is higher within one individual than between castes. Mean thickness is similar for workers and queens except for the propodeum of *Cataglyphis.*

### Redesign of the endoskeleton for optimized bracing and muscle attachment

**Fig. 3:**
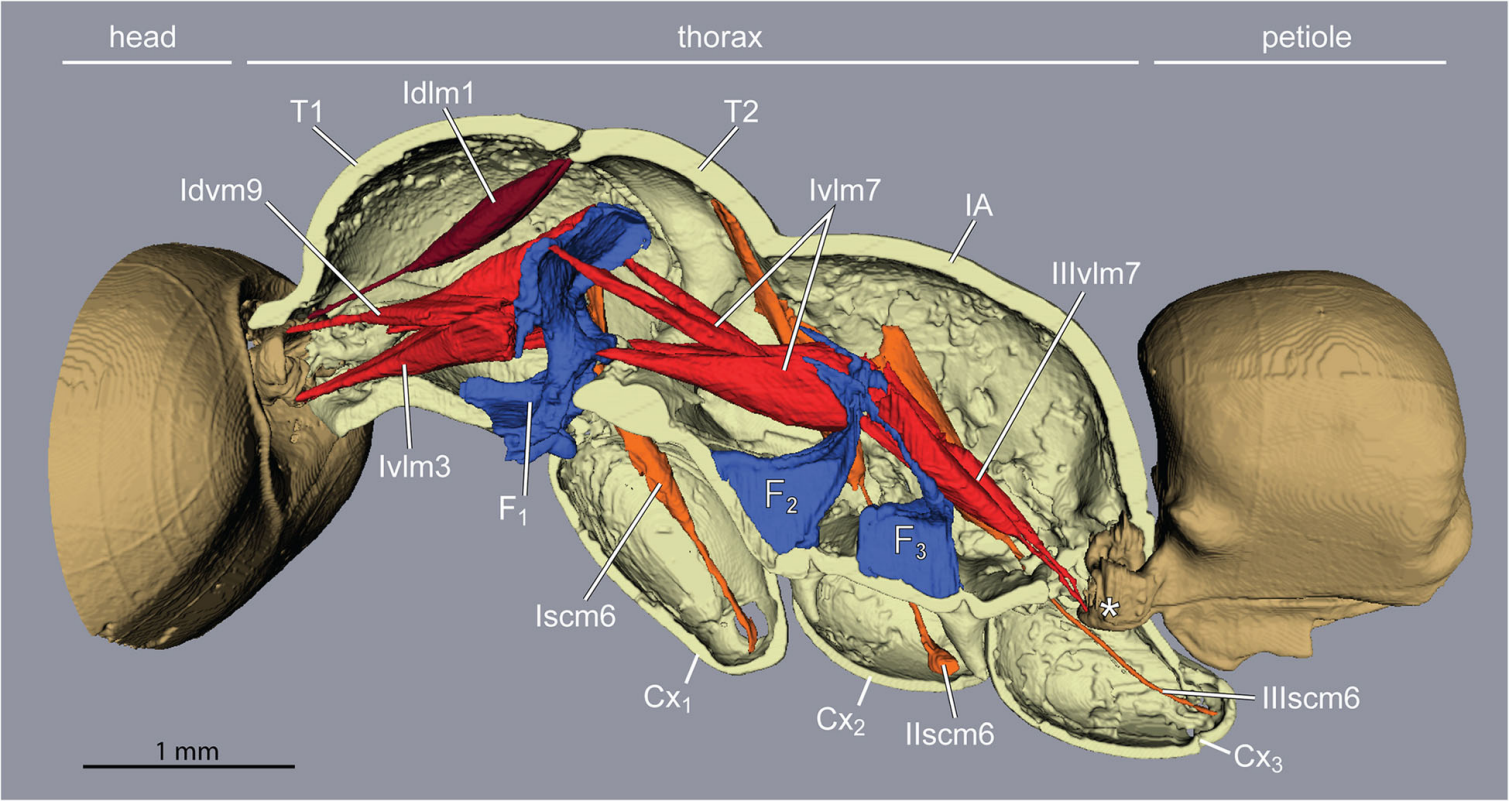
Tension muscles (red) connect head, furcae, and petiole along the thorax in ant workers (*Euponera sikorae*). Furcae (blue) are skeletal structures that relay force/stress inside the thorax. A direct muscle (Idlm1) originates dorsally at anterior edge of the mesonotum and pulls the head up. External trochanter muscles (orange, right legs only). Posteriorly, muscles arising on metafurca insert on the anterior end of the petiole, pulling the abdomen to maintain horizontality. T1, prothorax. T2, mesothorax. F_1_, profurca. F_2_, mesofurca. F_3_, metafurca. Cx_1_, Cx_2_ and Cx_3_ are coxae (= first segments) of the fore-, mid- and hindlegs respectively

**Fig. 4:**
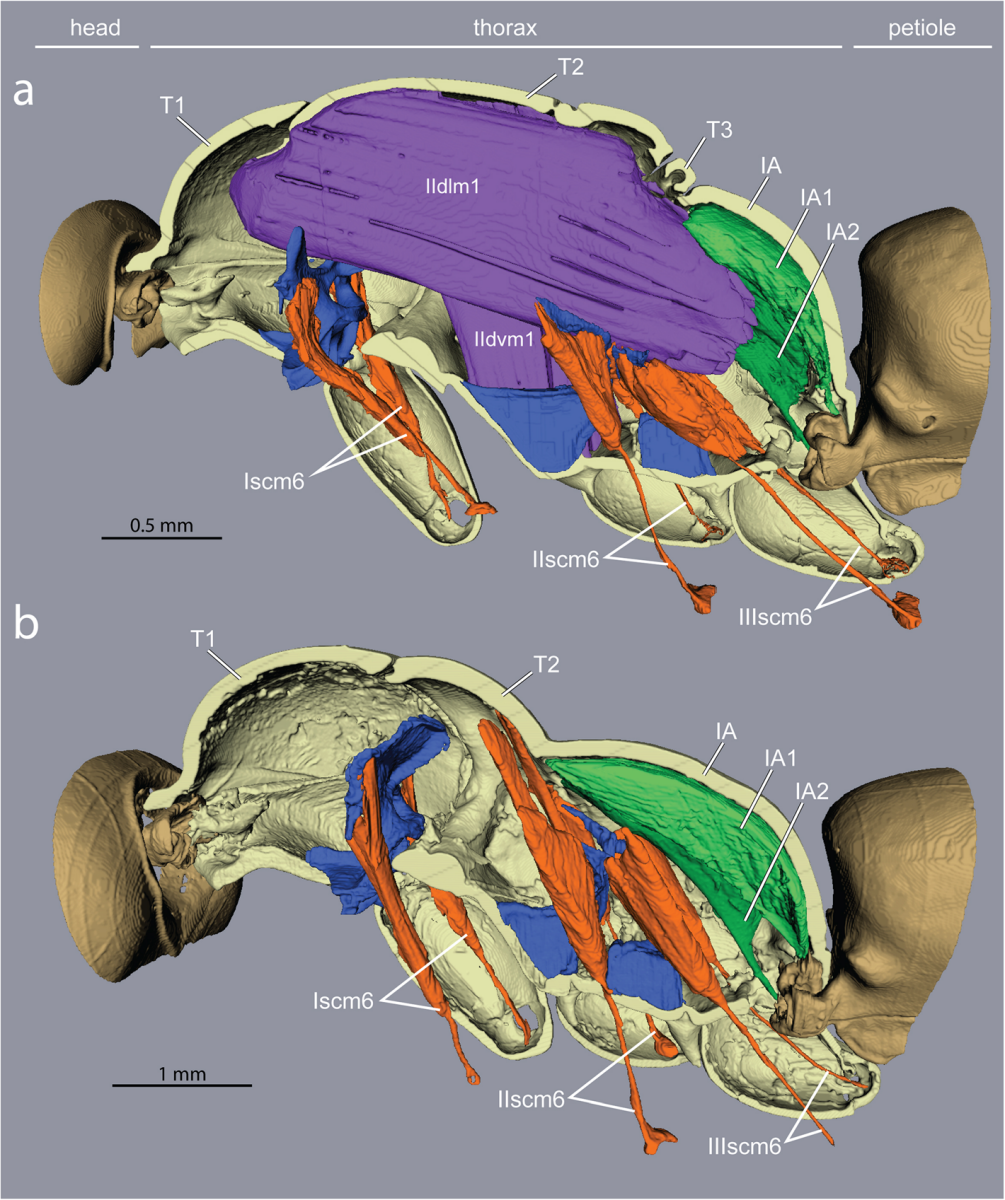
Impact of loss of wing muscles on geometry of leg muscles and petiole levators in *Euponera sikorae*. **a** In queens, wing muscles fill the thoracic cavity, limiting the size of leg and petiole muscles. **b** In workers, the external trochanter muscles (orange) of the midlegs (IIscm6) and hindlegs (IIIscm6) are extended to originate high on the roof of the mesothorax (T2) and the lateral walls of propodeum (IA) respectively, while the levators of the petiole (green) are larger and fill up almost the entire propodeal cavity

Inside the thoracic cavity, three invaginations of the *sternae* (ventral plates) rise up in front of each pair of legs, forming a row of rigid forked pillars*.* These *furcae* function as internal attachments for muscles that run between segments (head-to-prothorax, prothorax-to-mesothorax, metathorax-to-petiole), and muscles that move the legs (Fig. 3). In queens, the main stem of the furcae rise not more than one-third of the thorax’s height before bifurcating towards the sides (Fig. 4). This configuration results from the constraint of the huge longitudinal wing muscles that occupy the top two-thirds of the thoracic cavity (Fig. 1c and Fig. 2). In workers, the loss of wing muscles freed space within this cavity, allowing changes in size and shape of all three furcae.

The furcae of T2 and T3 are ancestrally fused into a single structure [18] (Fig. 3). The T2 furca bears a narrow transverse bridge that connects the distal points of its forked arms (Additional file 4). In winged Hymenoptera, these lateral arms connect with the walls of the upper thorax via a short muscle, providing the necessary flexibility for flight. In ant workers this muscular connection is lost, and we found that the T2 + 3 furcal arms are fused with the walls of the thorax, forming an internal bracing structure that possibly increases the overall rigidity of the thoracic box.


**Additional file 4 **Video showing how external trochanter muscles (orange) of mid- and hindlegs originate on the platform of furcae T2 and T3 (blue) in *Cataglyphis savignyi* queens. These furcae are merged into a single structure with the forked arms of the smaller T3 furca extending forward to fuse with the lateral arms of T2 furca. Depressor muscles of the petiole (light blue) originate on the base of T3 furca. 10.5281/zenodo.3406985.

In contrast with the short and fused T2 + 3 furcae, the profurca (T1) is larger and more elaborate across Hymenoptera. Both the median stem and the forked lateral arms are stout and support a sizeable bridge, shaped like a thin platform (Additional file 5). This platform provides attachment surfaces for three neck muscle pairs on its anterodorsal side (muscles Idvm9, Ivlm1, and Ivlm3) and a key foreleg muscle on its posteroventral side (see below). In flying queens, this platform is horizontal to allow the longitudinal wing muscles and oesophagus to run dorsally (discussed in Keller et al. 2014 [10]). Consequently, the neck muscles attach to the platform at a low angle. However, the lack of wing muscles in ant workers removes this constraint, and the platform takes a more vertical orientation resulting in a greater attachment surface and more favourable angles of origin for the large muscles that insert directly on the back of the head and support it (see below).

Unlike the T2 + 3 furcae, the profurca is not fused with the thorax walls, instead its median stem articulates freely in front of the foreleg insertions while its lateral arms connect to the thorax walls with membranes and a short muscle [8]. The neck muscles act antagonistically to both foreleg muscles (see below) and the intersegmental muscles (Ivlm7) that connect the profurca with T2 + 3 furcae (Fig. 3), transmitting stress from the head to all legs. Similarly, posture is maintained at the back of the thorax with intersegmental muscles (IIIvlm7) connecting T2 + 3 furcae to the petiole to transmit stress from the abdomen to the thoracic box.

### Reorganization of the neck muscles

The neck articulation in Hymenoptera is composed of four skeletal elements (Additional file 6): the *postocciput*, a short cup-shaped extension of the back of the head; a pair of triangular *propleura* that are ventrally situated in T1; and the profurca which sits inside T1. The head’s postocciput has a strong articulation with the thorax via a stiff neck membrane [22] as well as the anterior apodemes of the propleura [18]. The propleura are never fused to one another and these large plates can shift antagonistically for sideways and rotational movement of the head [23].

No muscles inside the head are involved in the movement of the head relative to the thorax. Rather, all neck muscles are prothoracic, moving the head either directly by inserting on the postocciput or indirectly by inserting on the propleura. In both queens and workers, four pairs of direct muscles lift the head up-and-down (Fig. 1c-d and Additional file 5). The lack of the anterior phragma in workers (associated with loss of wing muscles) affects especially muscle pair Idlm1. In queens, Idlm1 originates at the sides of their pliable phragma (Additional file 7), while in workers Idlm1 originates further back at the anterior margin of the roof of T2 (part of the rigid thoracic box; Fig. 3). Consequently, the better anchoring of muscle Idlm1, its extra length and lower angle of insertion to the postocciput (Additional file 7) give greater support and strength to the worker head in comparison to that of winged queens. The direct muscles Itpm1 originate at the base of propleura, hence their geometry differs little between queens and workers, but Itpm1 is relatively larger in workers for both species (Fig. 6). In contrast, muscle pairs Idvm9 and Ivlm3 are considerably affected by the queen-worker difference in the angle of the profurca platform (26° and 53° in *Euponera* queen and worker, respectively; 16° and 30° in *Cataglyphis* queen and worker; Additional file 5). In queens, as in wasps and bees, this platform is almost horizontal, and thus, these muscles have a very shallow angle to their origin. Contrary to queens, the upright inclination of the platform in workers allows a more favourable perpendicular angle of origin and a greater attachment surface for the equivalent muscles.

Two indirect muscle pairs are mostly responsible for sideways and rotational head movement by inserting on the anterior ends of the propleura. Ivlm1 originates on the profurca and thus benefits from the more favourable inclination of its platform in workers (Additional file 5). Idvm5 shows the most spectacular difference between queens and workers (Additional file 7). This large muscle pair originates at the pronotum (dorsal plate of T1) which is considerably larger across all worker ants. In addition to the increased area of attachment, the absence of wing muscles allows this muscle to support the propleura from a diagonal rather than a vertical angle. Both Idvm5 and Ivlm1 are relatively larger in workers for both species (Fig. 6).

### One crucial leg muscle differs between workers and flying queens

**Fig. 5:**
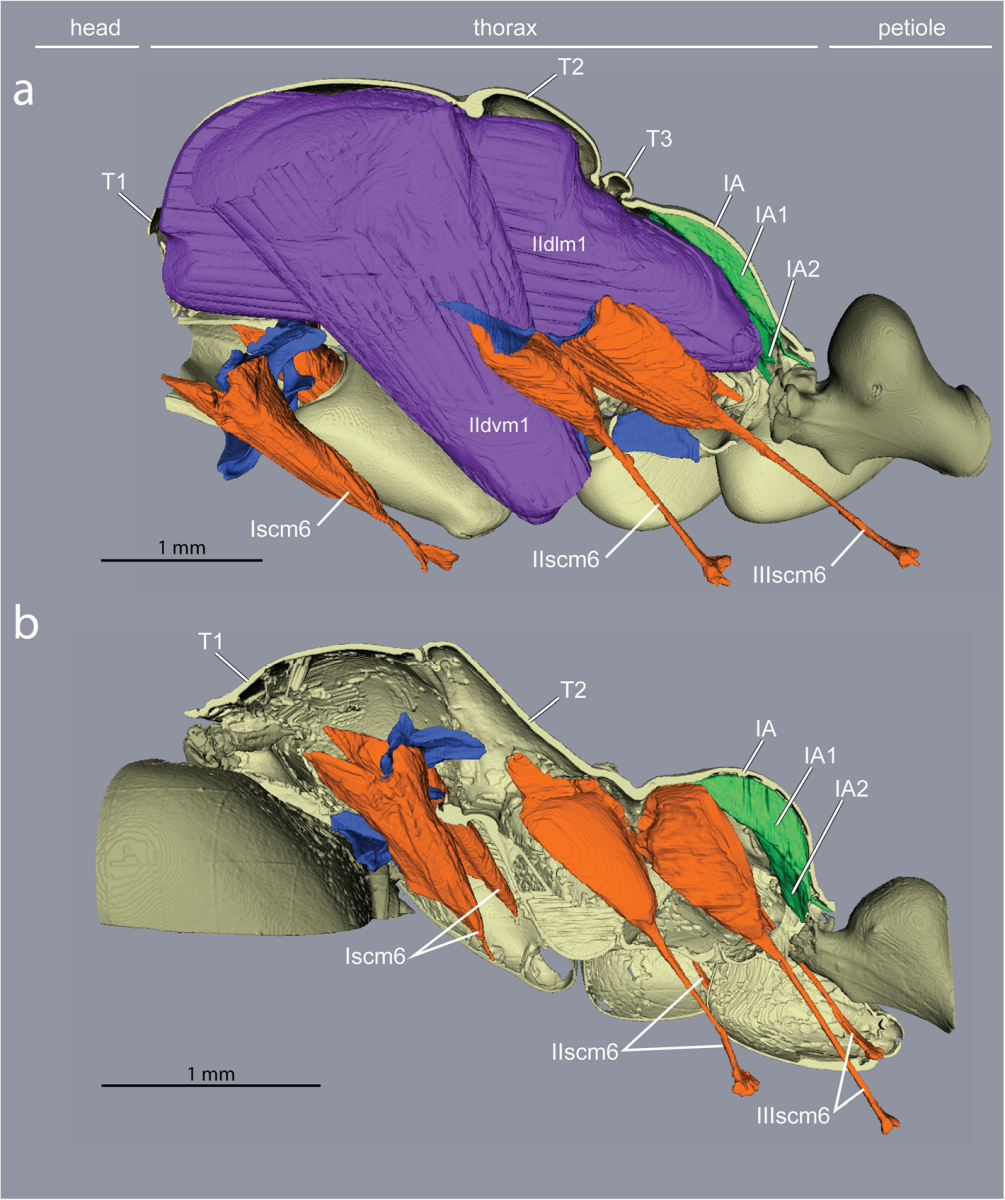
Loss of wing muscles and geometry of leg muscles and petiole levators in *Cataglyphis savignyi*. **a** In queens, wing muscles restrict both leg and petiole muscles. **b** In workers, the external trochanter muscles (orange) of the midlegs (IIscm6) and hindlegs (IIIscm6) are extended to originate high on the roof of the mesothorax (T2) and the lateral walls of propodeum (IA) respectively, while the levators of the petiole (green) increased in volume to fill up almost the entire propodeal cavity. T2/T3 furcae are not shown. Note thinner cuticle relative to *Euponera* (Fig. 4)

Back and forth movements of insect legs are controlled by a series of short muscles that originate within the thorax and insert around the opening of the *coxae*, the basalmost segment of each leg that articulates with the body. Leg flexion and extension, on the other hand, are controlled by muscles that reside entirely within the segments of the legs [18]. A notable exception is the external trochanter muscles. One per leg (Iscm6, IIscm6, IIIscm6), these muscles originate in the thorax but end in a long tendon that crosses the coxae and inserts on the *trochanter* (second leg segment) of all legs (Figs. 4 and 5). Contraction of the external trochanter muscles causes depression of the leg, lifting the body and carrying the ant’s weight plus any load. Because these muscles are housed outside the legs, with only their tendons crossing the coxae, their action is independent from the rotational motion of the coxae, and force is transmitted efficiently from the thorax to the legs no matter if walking or standing.

**Fig. 6:**
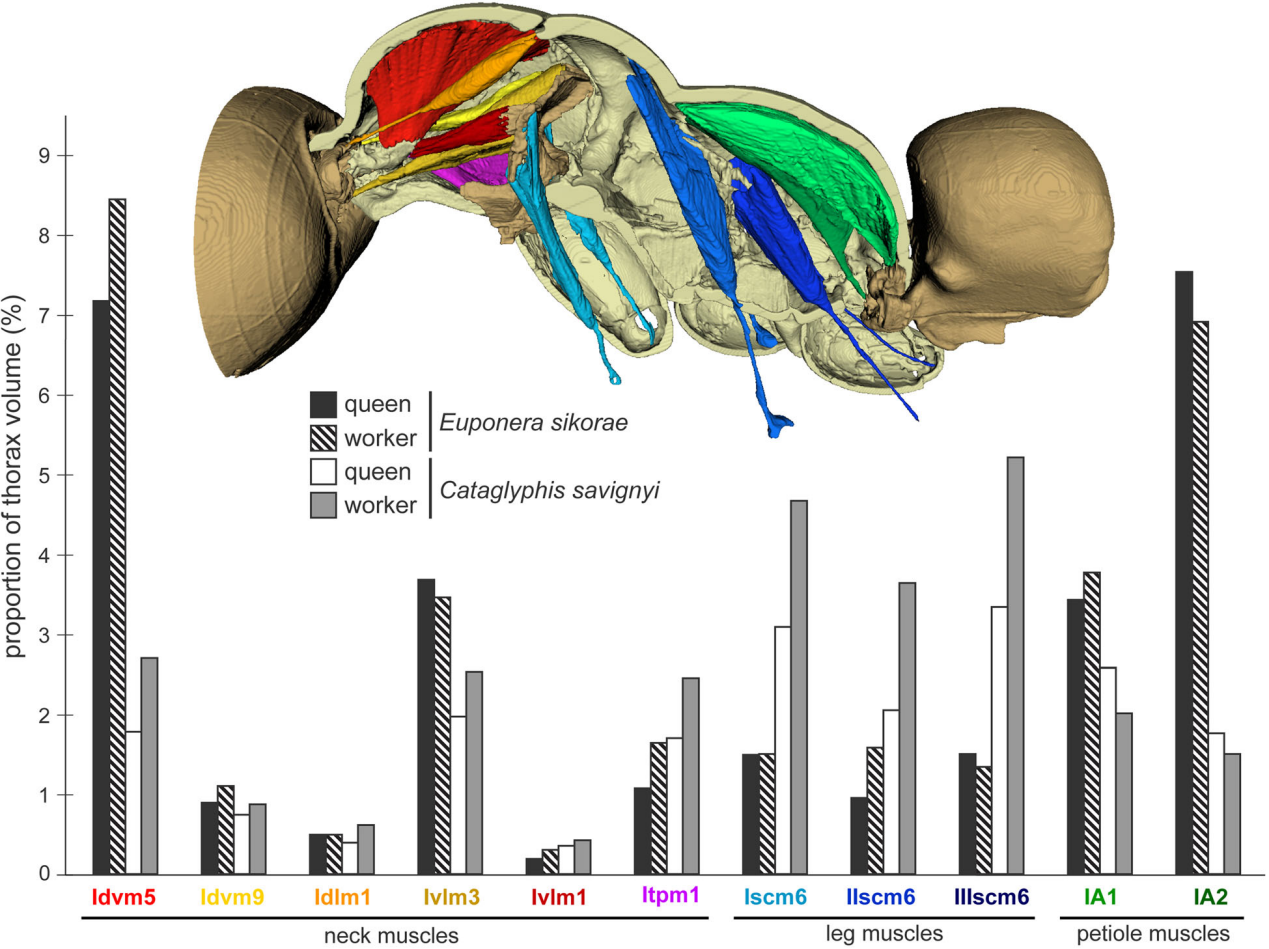
Differences in the proportion of neck, leg, and petiole muscles between worker and queen in *Euponera sikorae* and *Cataglyphis savignyi*. In *Cataglyphis*, neck and leg muscles are relatively larger in the worker, but petiole muscles are bigger in the queen. Measurements of muscle volumes were obtained from mCT scans (Additional file 9)

While the muscles of the coxae and those internal to the legs do not differ much between the castes, our segmentations show that the external trochanter muscles are very distinct in length, geometry, and place of origin between queens and workers. In queens, the external trochanter muscles are shorter because they originate on the furcal arms of their respective segment, just below the longitudinal wing muscles (Figs. 4 and 5). The absence of wing muscles in workers allows dorsal elongation of the mid and hind external trochanter muscles (IIscm6, IIIscm6), which attach either on the roof (*Cataglyphis*) or lateral walls (*Euponera*) of the rigid thoracic box (Figs. 4 and 5, Additional file 8). Queen-worker differences are minor for the forelegs: in both castes external trochanter muscles Iscm6 originate at the lower surface of the T1 furcal platform (Figs. 4 and 5). These muscles, therefore, act antagonistically to the neck muscles originating at the upper surface of this platform, helping to pull the head and transmitting force effectively from the head-neck articulation to the front legs. Workers in both species have a relatively larger IIscm6 than queens, and in *Cataglyphis* Iscm6 and IIIscm6 are also larger (Fig. 6).

### Enlargement of the petiole muscles

As with the head, all muscles responsible for articulating the free part of the abdomen are housed inside the thorax. Four muscle pairs (two dorsal, two ventral) insert on the anterior narrow end of the petiole, which protrudes in the back of the thorax. By acting antagonistically to each other, these muscles control both vertical and horizontal movements of the abdomen [18].

**Fig. 7:**
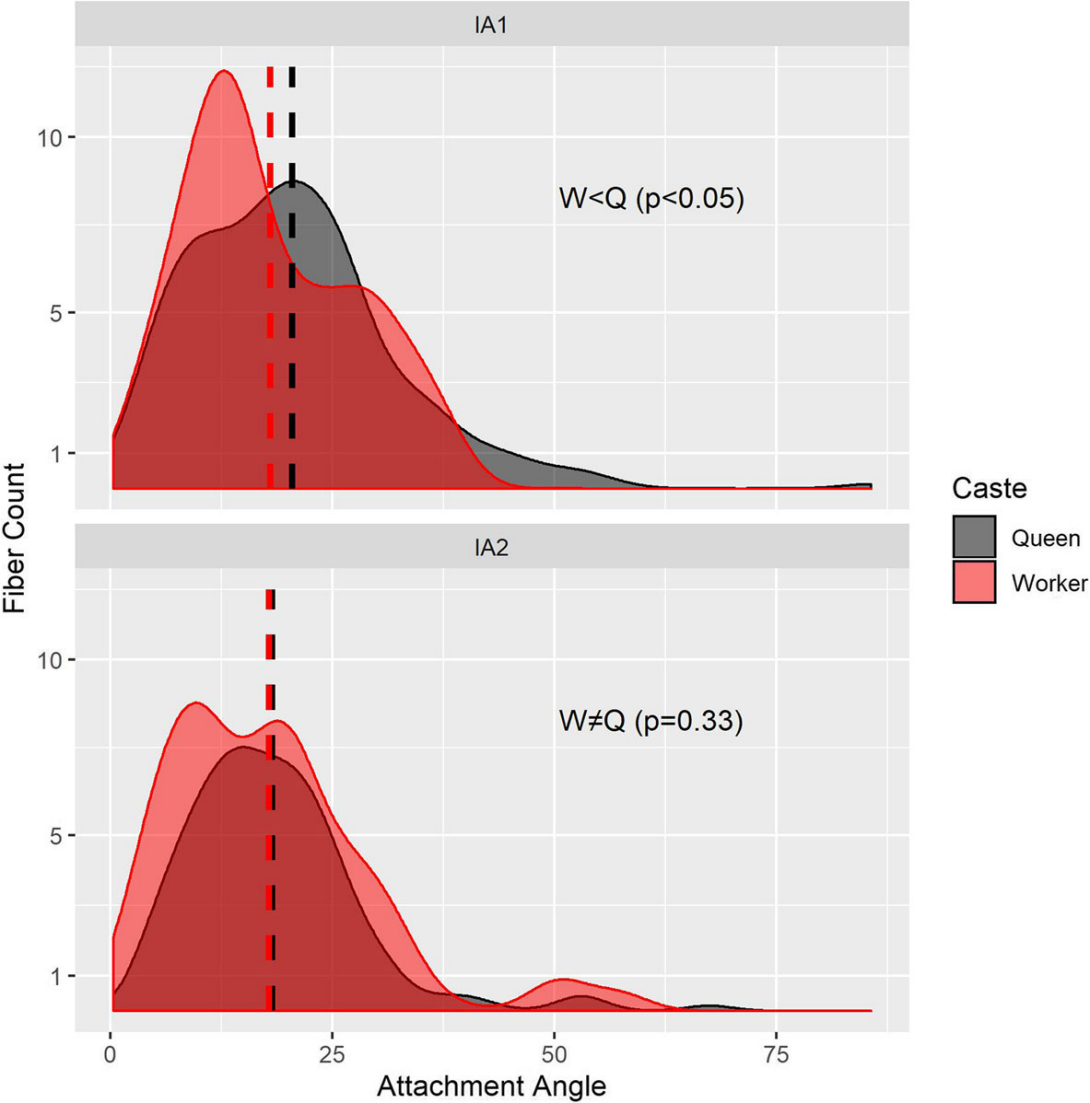
Distributions of fibre angles for the dorsal petiole muscles (top IA1; bottom IA2; see Additional file 8) differ between *Euponera sikorae* queen (grey) and worker (red). Fibres with an angle of 0° contract in the same direction as the force exerted by the muscle, hence lower angles mean more efficient fibres. Dashed line indicates mean angle. According to Mann-Whitney rank sum tests for non-normally distributed data, attachment angles in IA1 are significantly lower in worker than in the queen (W = 27,911, *p* < 0.05). There is no significant difference in distribution of angles between worker and queen in IA2 (W = 18,231, *p* = 0.33)

Our segmentations show that the two dorsal pairs differ strongly between queens and workers due to the presence/absence of the flight apparatus (Figs. 4 and 5). Muscles IA1 and IA2 originate on the roof of the propodeum (middle and lateral, respectively), but because of the posterior end of the horizontal wing muscles in queens, these petiole muscles are squeezed in the narrow space between the posterior phragma and the exoskeleton (Fig. 2). In workers, the lack of wing muscles means that muscles IA1 and IA2 fill most of the propodeal cavity (Figs. 4 and 5). Although relative muscle volumes are similar between queens and workers (Fig. 6), differences stand out in the fibre geometry in *E. sikorae*. First, IA1 has longer fibres in the worker than in the queen despite the smaller size of workers. In addition, IA1 fibres attach at significantly lower angles in the worker, resulting in greater efficiency (Fig. 7). Second, IA2 fibres are longer in the queen and attachment angles are not significantly different between worker and queen. However, the worker has 30% more muscle fibres with a broad distribution of angles, revealing how much this muscle can fan out in the absence of wing muscles.

Marked differences between the two different subfamilies are seen in muscles IA1 and IA2 of workers. In *Euponera* workers, these petiole muscles originate on the entire roof of the elongated propodeum (Fig. 4). In contrast, in the dome-shaped propodeum of *Cataglyphis,* the equivalent muscles do not extend forward and are relatively smaller in the worker than in the queen (Fig. 5); instead the anterior roof is the origin for the external trochanter muscles (IIIscm6) of the hind legs (Additional file 8).

### Comparison with other flightless insects

**Fig. 8:**
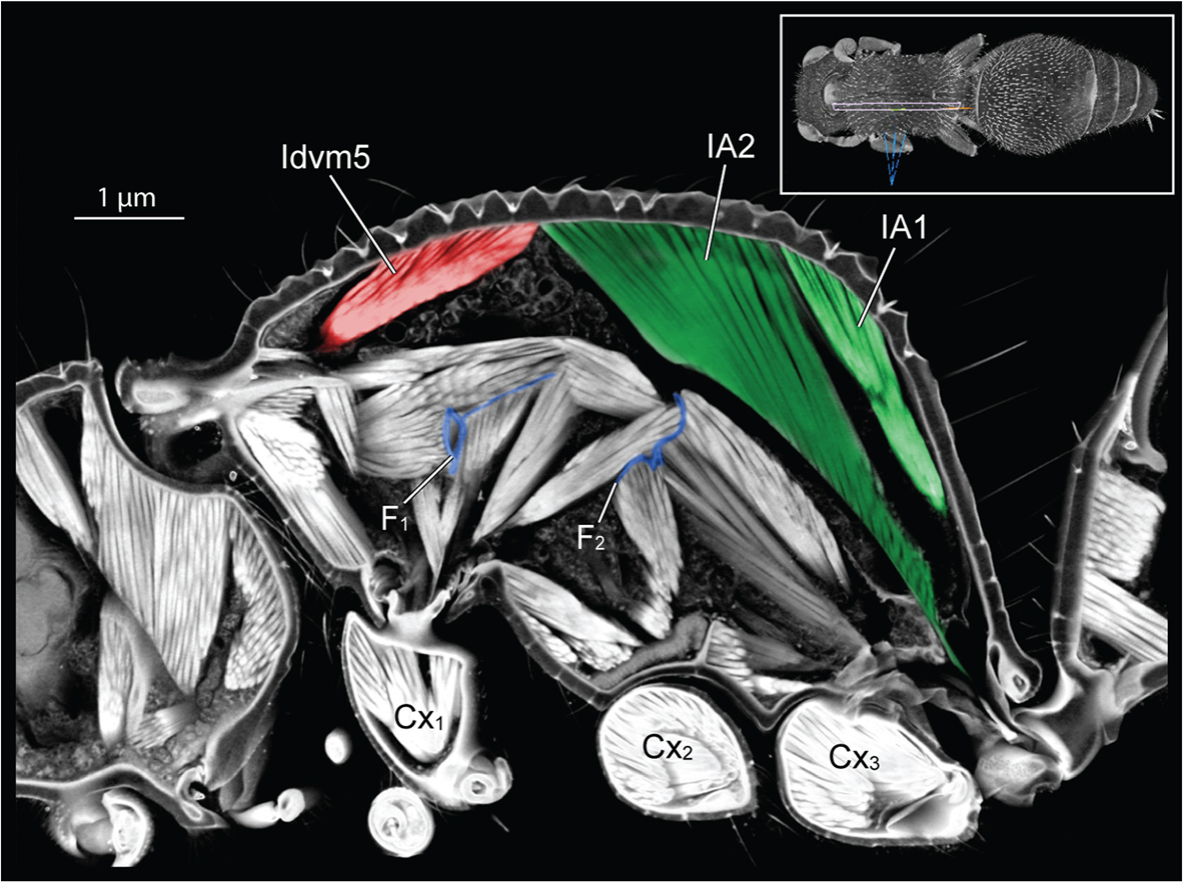
Three-dimensional section through the thorax of female mutillid (subfamily Mutillinae, tribe Smicromyrmini or Trogaspidiini). Inset (top right) indicates the plane of virtual sectioning. Note the greatly enlarged abdomen muscles (IA1 and IA2) that attach more anteriorly than in ant workers

Flight is the ancestral state across the insect tree of life, and its evolutionary loss occurred multiple times independently [24]. While a broad survey of flightlessness across insects is beyond the scope of this paper, we considered whether the modifications described here are specific to social as opposed to solitary lifestyles. In general, the lack of massive wing muscles does not lead to the same modifications of the thorax in solitary taxa. A female mutillid wasp shows strikingly enlarged abdomen muscles (there is no petiole), attached to most of the dorsal roof and reaching the anterior mesonotum (Fig. 8). This is congruent with the origin of the external trochanter of midlegs on T2 furca, instead of the propodeum roof. Given that mutillids are parasitoids that search for hosts underground and oviposit on the spot [25], this difference in thorax rearrangement likely reflects the absence of any selective pressures for prey transport. Indeed, added strength is not necessarily adaptive in solitary insects that are not central place foragers. In flightless moths (Lepidoptera) [26], ovaries reach into the posterior thorax, and the posterior phragma is retained to shield the ovaries; head muscles are not affected compared to flying males. Beetles (Coleoptera) fly with their hind wings only, hence flight musculature is confined to their enlarged T3 and does not conflict with the neck articulation. A functional approach makes it possible to interpret the internal adaptations associated with wing loss across insects showing divergent ground plans.

## Discussion

### Modifications in the thorax of wingless ant workers

Our micro-CT comparison of queens and workers in two distant subfamilies reveals that the evolutionary loss of the flying engine typical of insects allowed for a remodelling of the thoracic skeleton and associated muscles to power earthbound activities. We showed worker-specific modifications in the three endoskeletal furcae that are key for muscle attachment as well as transmission of load between mandibles, legs and abdomen. Our measures of muscle volumes and pennation angles confirmed the conspicuous pattern of repositioning and enlargement seen in 3D segmentations. We highlight the importance of indirect muscles in the prothorax that move the head sideways by shifting two ventral plates (propleura). These adaptations for strength likely result from the selective pressures of central place foraging, that necessitates carrying or dragging food to the nest. Strength is not a general feature of flightless insects but rather an adaptation for maximizing the acquisition of resources for ant colonies. In contrast, solitary mutillid wasp females use vacant space in the thorax for much enlarged abdomen muscles, allowing for better performance as a stinging parasitoid.

Our comparative survey across phylogenetically disparate species revealed a consistent pattern of expanded muscles with altered geometry (Table 1). Although all ant workers have a similarly modified thorax relative to queens, there are minor differences reflecting trade-offs in abilities across lineages. Many ponerines, especially “tank-like” ones such as *Euponera,* need more strength to manoeuver the sting and hold the heavy abdomen level. By contrast, formicines like *Cataglyphis* are highly gracile, fast-running ants with a relatively small gaster, thin cuticle, and they lack a sting. While *Euponera* workers use the expanded propodeal roof for petiole muscles only (IA1 and IA2, Fig. 4), in *Cataglyphis* the more dome-shaped propodeum is used as attachment surface for both the petiole and hindleg muscles (Fig. 5). Our m-CT segmentations of *Euponera* and *Cataglyphis* queens confirm earlier reports [10] of differences in neck muscles contingent on the need to forage or not during colony foundation; the prothorax of non-claustral *Euponera* queens is more similar to workers’ (Additional file 1, Figs. 4 and 5).

### Functional morphology of the thorax in ants

We describe here the morphological redesign of the thorax, and present hypotheses about its functional implications, but it remains a challenge to directly link morphology with performance. A comprehensive modeling effort focusing on the ant thorax is needed to definitively link structure and function, but this presents a formidable challenge. Although we measured the volumes and angles for a few muscles, mathematical analysis of how these changes relate to greater strength or faster contraction speed remains difficult in insects. Moreover, cuticle parameters such as thickness and elasticity need to be integrated in the analysis, but our results indicate that cuticle thickness changes across different regions of an individual thorax. Hence simplifying thickness to single values for each individual is a source of errors that complicates mathematical models. Future studies need to refine the biomechanical connection between skeletomuscular specialization of each subsystem (neck, legs and petiole) and overall strength.

The ability to carry heavy prey or liquid food, and to manipulate objects, allows a broader range of potential food items (arthropods live or dead, seeds, honeydew or other sweet secretions) and nest constructions. While activities inside the nests may be less reliant on athletic abilities, foraging encompasses carrying and dragging food, running to escape predators, and the ability to defend and hunt (e.g. mandible dexterity, stinging or formic acid spraying). Given that ant queens retain the ability to disperse by flight in most species, workers were free to combine morphological adaptations for ground labour and social skills. So many benefits derive from the loss of flight in ant workers that, until now, musculoskeletal modifications have been overlooked. In addition to obvious energy savings related to lost wing muscles, both manufacture and maintenance [27], the loss of flight in workers was a decisive innovation that revolutionized colonial economy - cheaper workers allow for more populous colonies whenever this is adaptive [28].

## Conclusions

The attributes of individual phenotypes are likely just as crucial to the evolution of social life as genetic caste differentiation mechanisms, chemical communication, kin-recognition systems, and collective behaviour algorithms. Both morphology and social behaviour need to be integrated to contrast the colonial benefits of having wingless workers in ants as opposed to winged workers in social bees and wasps. All worker castes in Hymenoptera evolved specializations for tasks complementary to those of the queen caste, but the chasm is much less dramatic in social wasps and bees because the workers need to fly. Our insights in the adaptations of a thorax rearranged for strength on six legs reveal that ants maximized the merits of having queen and worker castes, allowing for a much greater divergence in body size compared to social bees and wasps. We propose this redesign of the worker phenotype for ground labour combined with the benefits of sociality is key to understanding the ecological success of ants.

## Supplementary information

**Additional file 1 **3D reconstruction of the thorax of *Euponera sikorae* queen (A) and worker (B). Note that the pronotum is almost the same size in both castes, unlike in *Cataglyphis* (Fig. 1).

**Additional file 2 ***Cataglyphis savignyi* worker, sides and dorsum compressed to give mid-thorax the appearance of an hourglass.

**Additional file 3 **Measurements of cuticle thickness for *Cataglyphis* and *Euponera* queens and workers. Using virtual slices through the pronotum (purple) and propodeum (green) of 3D models, cuticle thickness was measured at ten different locations, labelled 1 to 10. Values (in μm) for each location and specimen are shown in the Table.

**Additional file 5 **Geometry of neck muscles in prothorax of *Cataglyphis savignyi* queen versus worker, showing attachments with postocciput (left) and platform of profurca (right). A-D, dorsal view. E-G, lateral view. In E-F, the highest (a) and lowest (b) points of the platform were used to measure platform inclination (PI). Scale bars = 0.5 mm.

**Additional file 6 **Skeletal neck articulation of *Cataglyphis savignyi* queen. (A) Ventral view. (B) Lateral view. Pronotum (dorsal) not shown.

**Additional file 7 **Ventral views of prothorax showing neck muscles in queens and workers of *Euponera sikorae* and *Cataglyphis savignyi.* Loss of flight muscles (dark blue) allows expansion and reorientation of direct muscle Idlm1 (orange) and indirect muscle Idvm5 (red).

**Additional file 8 **(A) Dorsal view of 3D reconstruction of muscles attached on the propodeum and posterior furcae of *Cataglyphis savignyi* worker. (B) and (C) Transverse 2D cross-sections at anterior and posterior planes. Furcae T2 + T3 in blue, external trochanter IIscm6 in salmon, external trochanter IIIscm6 in red, coxa muscle in yellow, petiole muscle IA1 (levator) dark green, IA2 (sideways movement) light green, IIIvlm2 (depressor) pale green.

**Additional file 9 **: **Table S1.** Muscle volumes in worker and queen of *Euponera sikorae* and *Cataglyphis savignyi*. Muscle volumes were normalized using the inner thorax volume (after excluding the volume of wing muscles in queens) to compare allometry between workers and simplified (wingless) queens. Wing muscles represent 41 and 52% of the inner thorax in *E. sikorae* and *C. savignyi* respectively. Ratios superior to 1 indicate hyperallometry in the worker.

## Data Availability

All datasets are deposited on Zenodo https://dx.doi.org/xxxx
