# Supplementary figures and images for: The loss of flight in ant workers enabled an evolutionary redesign of the thorax for ground labour
Source: Front Zool. 2020 Oct 19;17:33. doi: 10.1186/s12983-020-00375-9 (PMC7574298; doi:10.1186/s12983-020-00375-9)

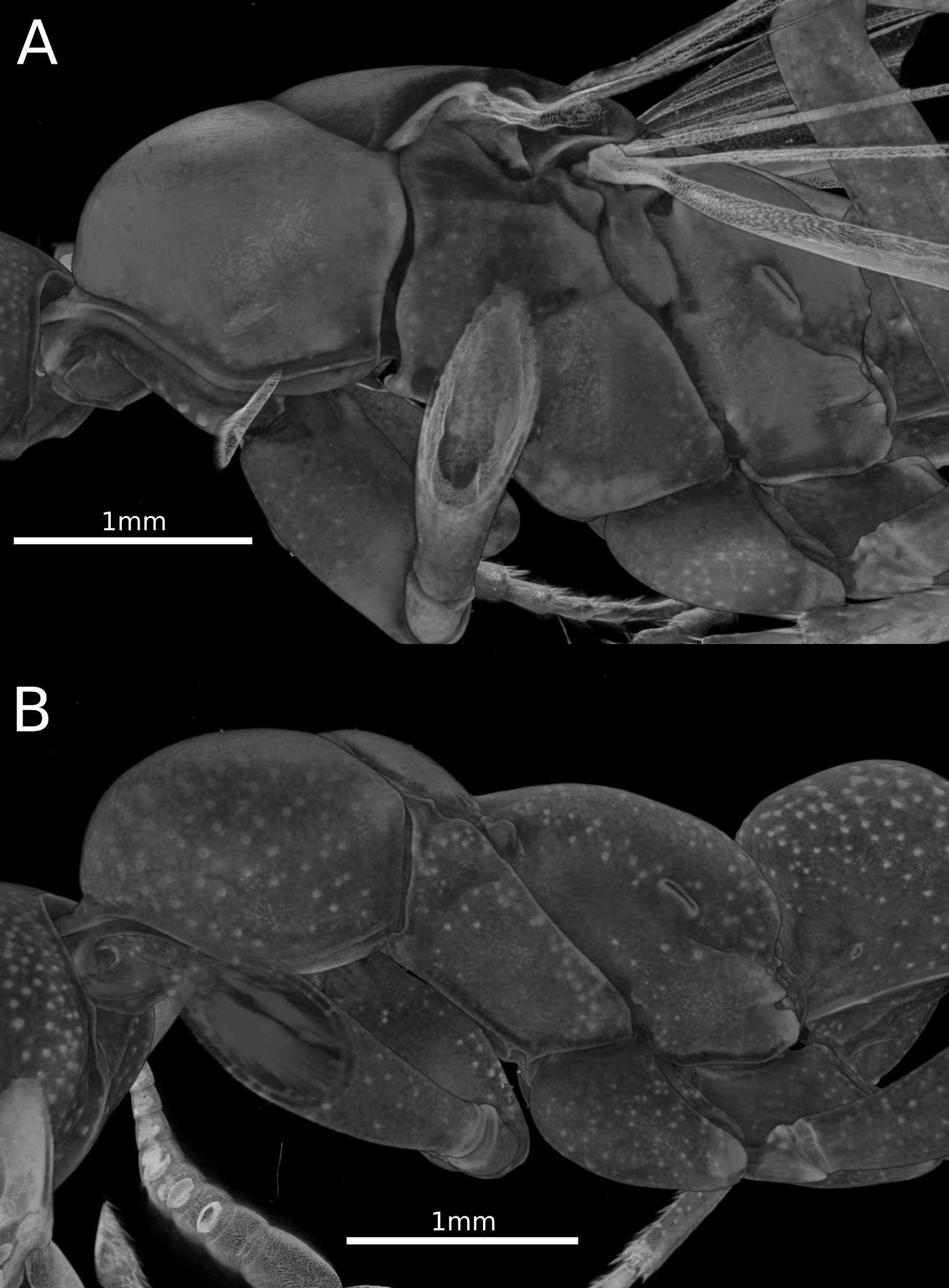

Supplement: Supplementary file 2 — Additional file 1 3D reconstruction of the thorax of Euponera sikorae queen (A) and worker (B). Note that the pronotum is almost the same size in both castes, unlike in Cataglyphis (Fig. 1). [file 12983_2020_375_MOESM1_ESM.png]

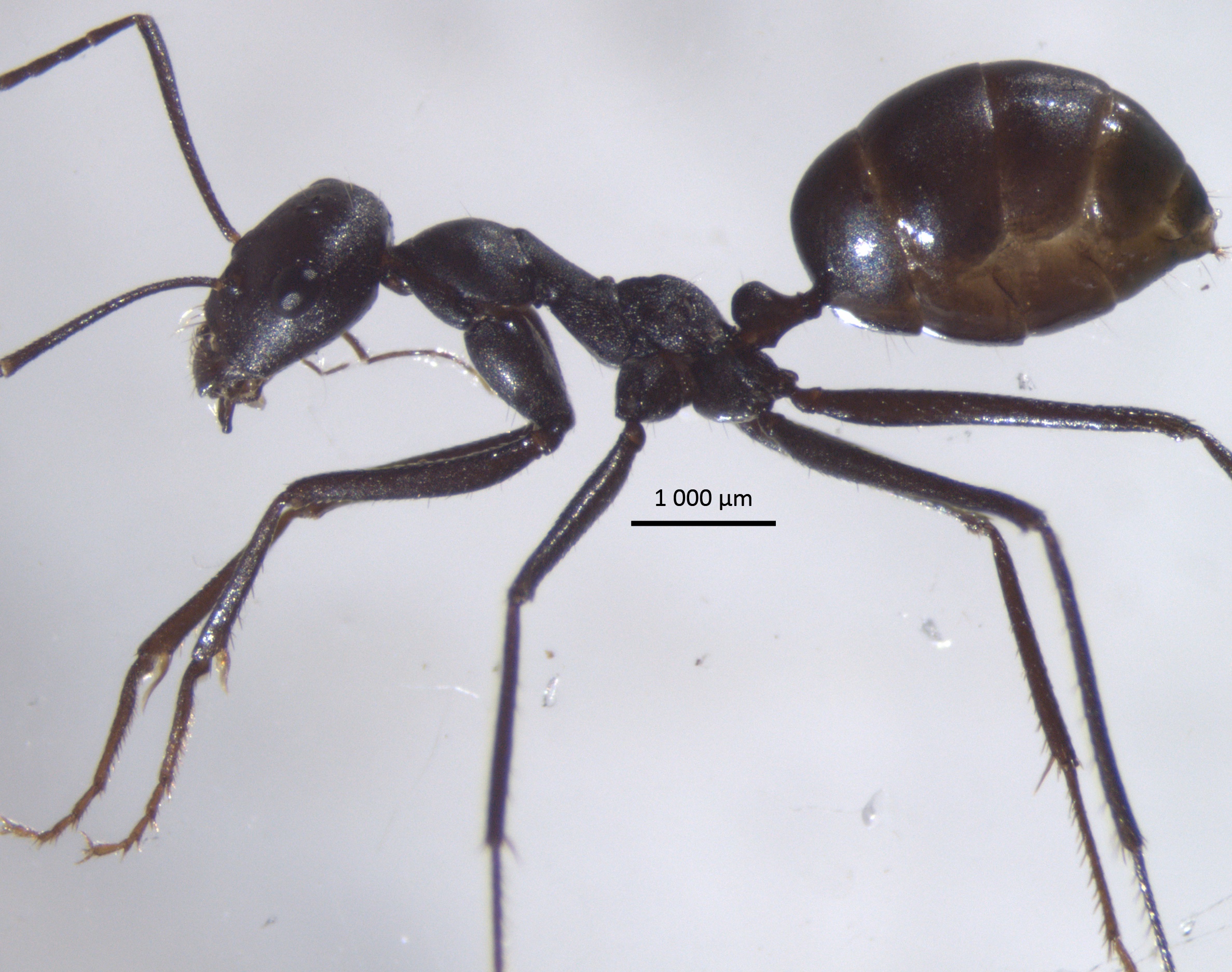

Supplement: Supplementary file 3 — Additional file 2 Cataglyphis savignyi worker, sides and dorsum compressed to give mid-thorax the appearance of an hourglass. [file 12983_2020_375_MOESM2_ESM.jpg]

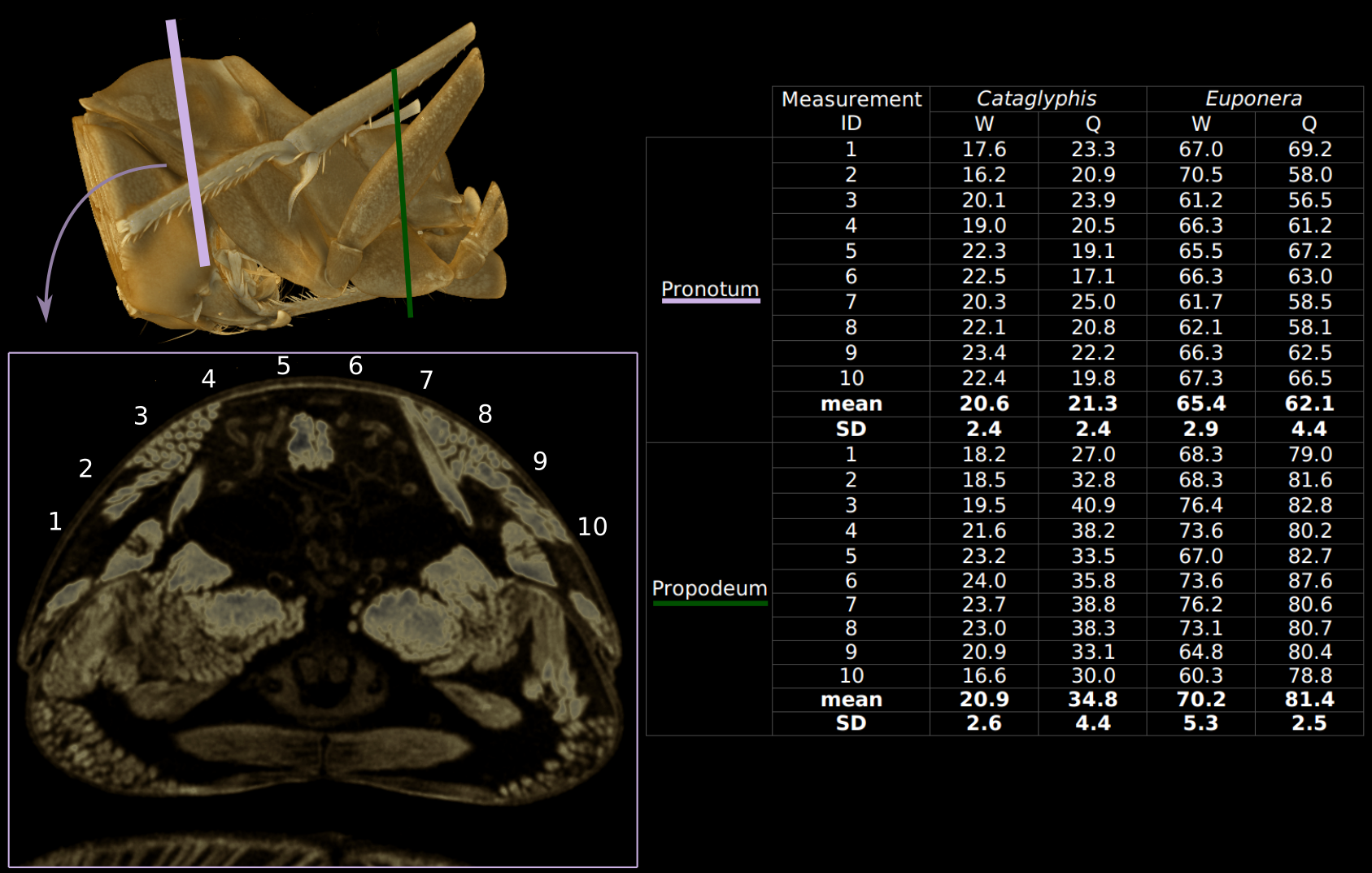

Supplement: Supplementary file 4 — Additional file 3 Measurements of cuticle thickness for Cataglyphis and Euponera queens and workers. Using virtual slices through the pronotum (purple) and propodeum (green) of 3D models, cuticle thickness was measured at ten different locations, labelled 1 to 10. Values (in μm) for each location and specimen are shown in the Table. [file 12983_2020_375_MOESM3_ESM.png]

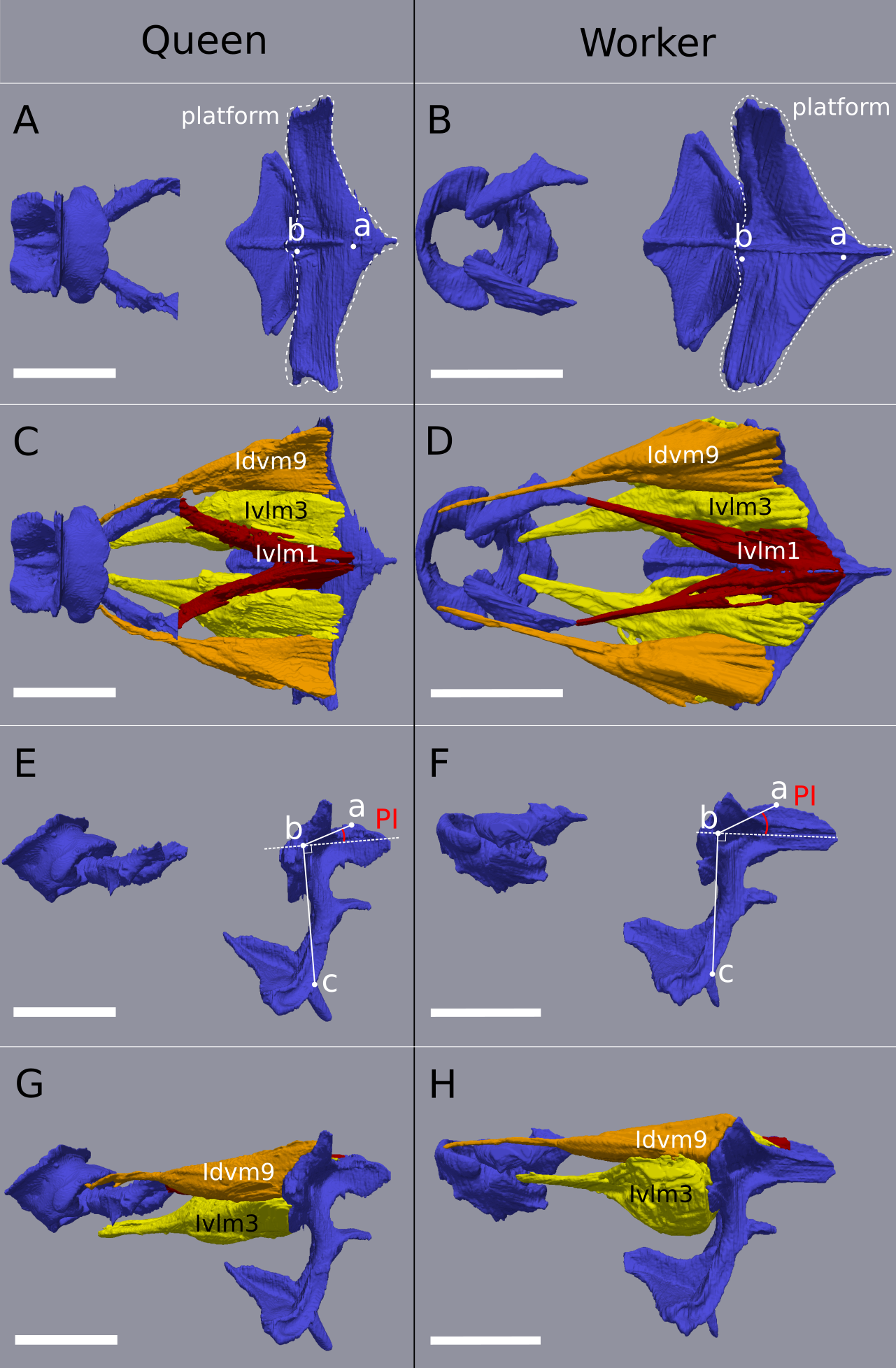

Supplement: Supplementary file 5 — Additional file 5 Geometry of neck muscles in prothorax of Cataglyphis savignyi queen versus worker, showing attachments with postocciput (left) and platform of profurca (right). A-D, dorsal view. E-G, lateral view. In E-F, the highest (a) and lowest (b) points of the platform were used to measure platform inclination (PI). Scale bars = 0.5 mm. [file 12983_2020_375_MOESM5_ESM.png]

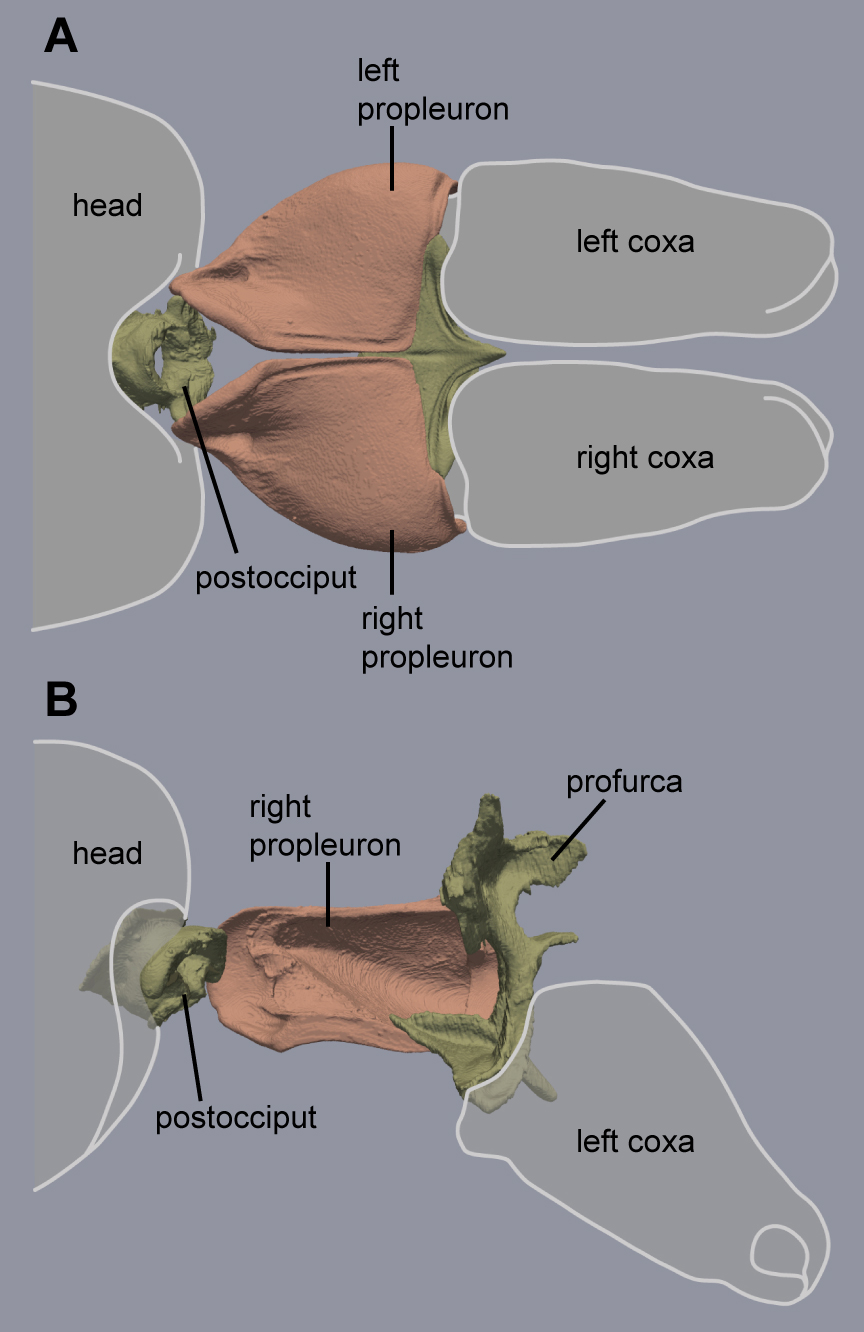

Supplement: Supplementary file 6 — Additional file 6 Skeletal neck articulation of Cataglyphis savignyi queen. (A) Ventral view. (B) Lateral view. Pronotum (dorsal) not shown. [file 12983_2020_375_MOESM6_ESM.jpg]

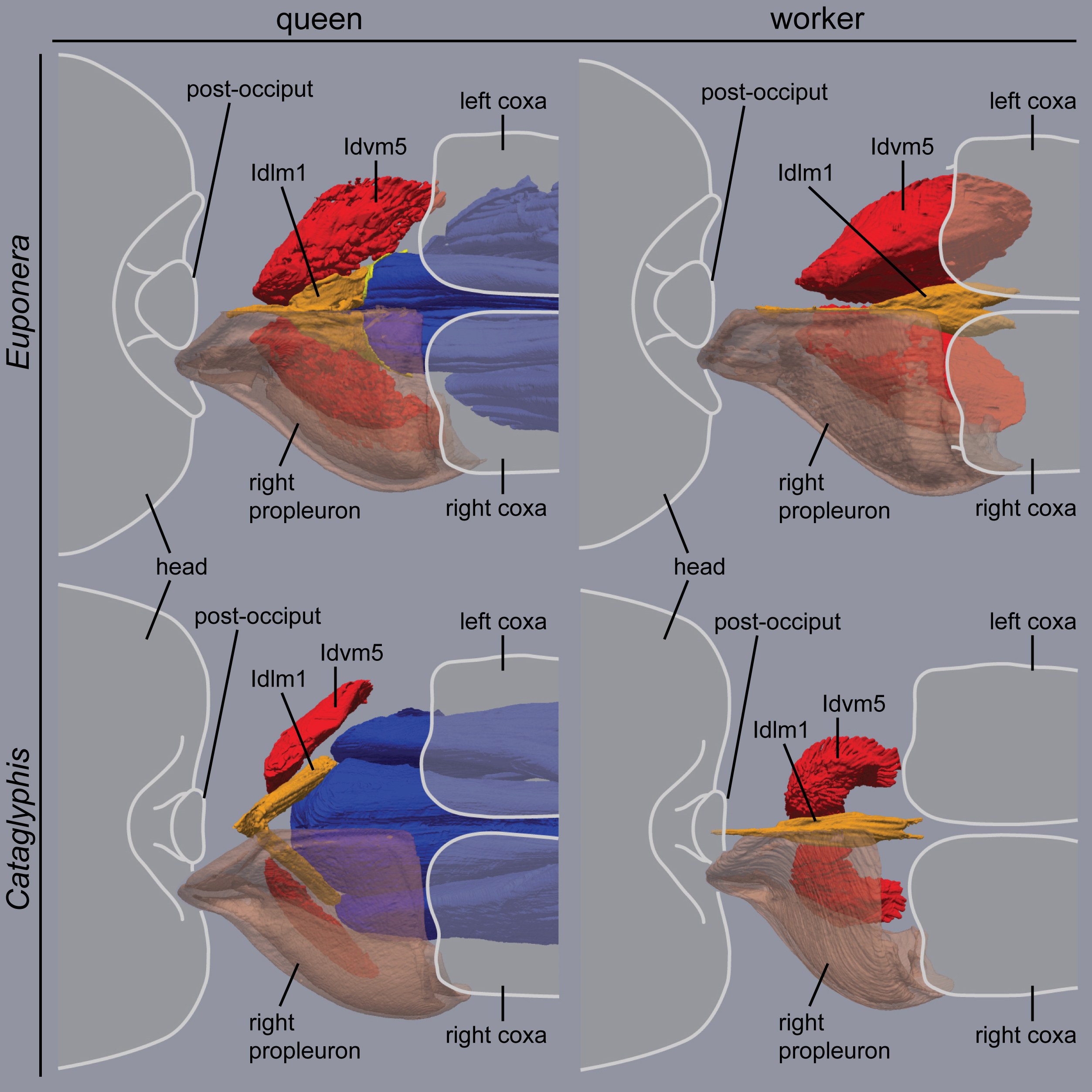

Supplement: Supplementary file 7 — Additional file 7 Ventral views of prothorax showing neck muscles in queens and workers of Euponera sikorae and Cataglyphis savignyi. Loss of flight muscles (dark blue) allows expansion and reorientation of direct muscle Idlm1 (orange) and indirect muscle Idvm5 (red). [file 12983_2020_375_MOESM7_ESM.jpg]

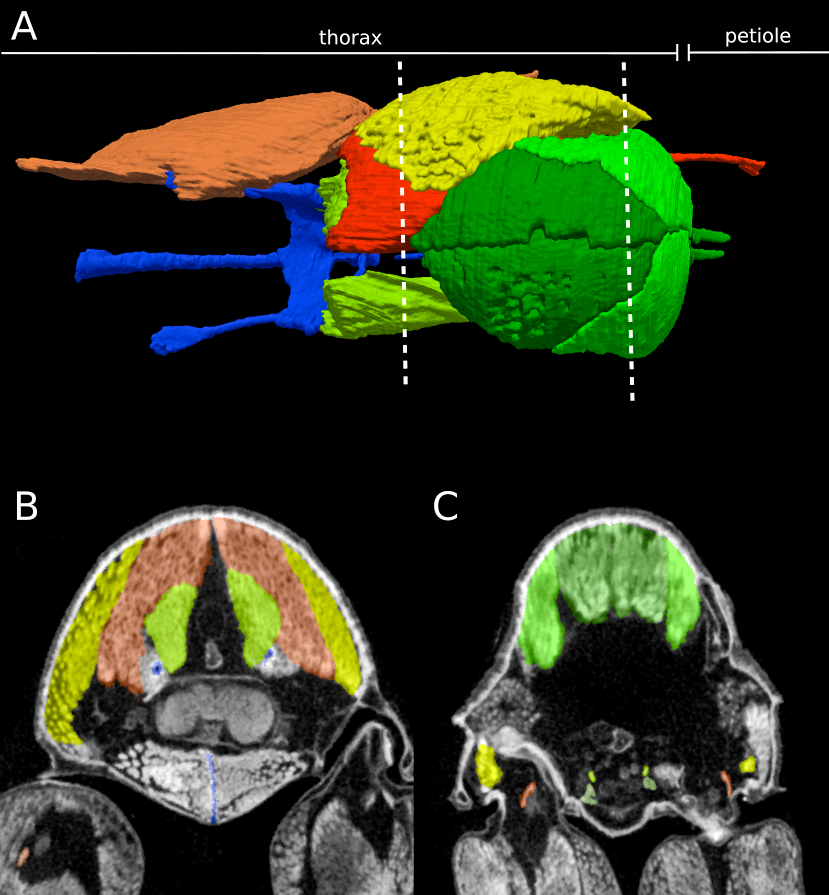

Supplement: Supplementary file 8 — Additional file 8 (A) Dorsal view of 3D reconstruction of muscles attached on the propodeum and posterior furcae of Cataglyphis savignyi worker. (B) and (C) Transverse 2D cross-sections at anterior and posterior planes. Furcae T2 + T3 in blue, external trochanter IIscm6 in salmon, external trochanter IIIscm6 in red, coxa muscle in yellow, petiole muscle IA1 (levator) dark green, IA2 (sideways movement) light green, IIIvlm2 (depressor) pale green. [file 12983_2020_375_MOESM8_ESM.png]
