# Supplementary material for: The loss of flight in ant workers enabled an evolutionary redesign of the thorax for ground labour
Source: Front Zool. 2020 Oct 19;17:33. doi: 10.1186/s12983-020-00375-9 (PMC7574298; doi:10.1186/s12983-020-00375-9)
Supplement: Supplementary file 9 — Additional file 9 : Table S1. Muscle volumes in worker and queen of Euponera sikorae and Cataglyphis savignyi. Muscle volumes were normalized using the inner thorax volume (after excluding the volume of wing muscles in queens) to compare allometry between workers and simplified (wingless) queens. Wing muscles represent 41 and 52% of the inner thorax in E. sikorae and C. savignyi respectively. Ratios superior to 1 indicate hyperallometry in the worker. [file 12983_2020_375_MOESM9_ESM.docx]

|  |  |  | ***Euponera sikorae*** | | | ***Cataglyphis savignyi*** | | |
| --- | --- | --- | --- | --- | --- | --- | --- | --- |
|  |  | Muscle ID (from Liu et al.) | Volume in µm³ (**proportion**) in worker | Volume in µm³ (**proportion**) in queen | Ratio of proportions worker/queen | Volume in µm³ (**proportion**) in worker | Volume in µm³ (**proportion**) in queen | Ratio of proportions worker/queen |
| inner thorax without wing muscles | |  | 2411 (**100%**) | 3627 (**100%**) | 1 | 3365 (**100%**) | 6244 (**100%**) | 1 |
| head muscles | direct | Idlm1 | 12 (**0.5%**) | 18 (**0.5%**) | 1 | 20 (**0.6%**) | 24 (**0.4%**) | 1.6 |
|  |  | Itpm1 | 40 (**1.7%**) | 39 (**1.1%**) | 1.5 | 82 (**2.5%**) | 106 (**1.7%**) | 1.4 |
|  |  | Idvm9 | 27 (**1.1%**) | 32 (**0.9%**) | 1.2 | 29 (**0.9%**) | 46 (**0.7%**) | 1.2 |
|  |  | Ivlm3 | 84 (**3.5%**) | 133 (**3.7%**) | 0.9 | 85 (**2.5%**) | 123 (**2.0%**) | 1.3 |
|  | indirect | Idvm5 | 205 (**8.5%**) | 260 (**7.2%**) | 1.2 | 91 (**2.7%**) | 111 (**1.8%**) | 1.5 |
|  |  | Ivlm1 | 7 (**0.3%**) | 7 (**0.2%**) | 1.6 | 14 (**0.4%**) | 22 (**0.4%**) | 1.2 |
| external trochanter muscles | leg 1 | Iscm6 | 36 (**1.5%**) | 54 (**1.5%**) | 1.0 | 157 (**4.7%**) | 193 (**3.1%**) | 1.5 |
|  | leg 2 | IIscm6 | 38 (**1.6%**) | 35 (**1.0%**) | 1.7 | 123 (**3.6%**) | 128 (**2.1%**) | 1.8 |
|  | leg 3 | IIIscm6 | 33 (**1.4%**) | 54 (**1.5%**) | 0.9 | 175 (**5.2%**) | 208 (**3.3%**) | 1.6 |
| petiole muscles | | IA1 | 92 (**3.8%**) | 124 (**3.4%**) | 1.1 | 67 (**2.0%**) | 161 (**2.6%**) | 0.8 |
|  |  | IA2 | 168 (**7.0%**) | 274 (**7.5%**) | 0.9 | 51 (**1.5%**) | 110 (**1.8%**) | 0.8 |

Table S1: Muscle volumes in worker and queen of *Euponera sikorae* and *Cataglyphis savignyi*. Muscle volumes were normalized using the inner thorax volume (after excluding the volume of wing muscles in queens) to compare allometry between workers and simplified (wingless) queens. Wing muscles represent 41% and 52% of the inner thorax in *E. sikorae* and *C. savignyi* respectively. Ratios superior to 1 indicate hyperallometry in the worker.
